# Comparison of two nutritional risk screening tools in hospitalized children with Japanese encephalitis: a causal inference of clinical outcomes and implications for optimized management

**DOI:** 10.1186/s13052-025-01922-y

**Published:** 2025-03-22

**Authors:** Yilei Shen, Lijuan Xu, Tian Tan, Wei Cao, Yong Zhao, Yue Feng, Xia Li, Yongfang Liu, Yingting Luo, Lin Kong

**Affiliations:** 1 School of Mathematics, Sichuan University, Chengdu, 610065 China; 2 Department of Clinical Nutrition, Children’s Hospital of Chongqing Medical University, Chongqing, China; 3 National Clinical Research Center for Child Health and Disorders, Chongqing, China; 4 Ministry of Education Key Laboratory of Child Development and Disorders, Chongqing, China; 5 Chongqing Key Laboratory of Child Rare Diseases in Infection and Immunity, Chongqing, China; 6 Chinese Center for Disease Control and Prevention, National Institute for Nutrition and Health, Beijing, China; 7 Key Laboratory of Public Nutrition and Health, National Health Commission of the People’s Republic of China, Beijing, 100050 China; 8 Nutrition Innovation Platform-Sichuan and Chongqing, Chongqing, China; 9 Science Popularization Studio, Chongqing, China

**Keywords:** Children, Japanese encephalitis, Nutritional risk, Clinical outcomes, Causal analysis, Decision tree (machine learning)

## Abstract

**Background:**

This study used two nutritional risk screening (NRS) tools to explore the causal relationship between nutritional risk and clinical outcomes (length of hospital stay and cost), as well as clinical results (incidence of sequelae), in hospitalized children with Japanese encephalitis (JE). The goal is to screen for a more suitable nutrition risk tool for JE reveal the underlying mechanisms, accurately quantify the impact, and provide a reliable basis for optimizing clinical management and reducing the burden of the disease in affected children.

**Methods:**

The classical Screening Tool for Risk of Nutrition in Growth Kids (STRONGkids) and Screening Tool for Assessment of Malnutrition in Pediatrics (STAMP) were utilized to evaluate the nutritional risk of the children. A heatmap analysis was conducted to investigate the correlation between variables influencing the STRONGkids score and STAMP score. Subsequently, a decision tree was employed to identify the main factors influencing the STRONGkids score and STAMP score. Finally, causal inference was employed to calculate the causal effects between the NRS score, clinical outcomes, and clinical results.

**Results:**

Dysphagia was the most significant factors affecting STRONGKids scores, and the weight and height was the most significant factors affecting STAMP scores. Causal analysis revealed that for every unit increase in the severity of JE type, the STRONGkids score increased by 0.515 units, and 1.339 units for STAMP. Moreover, the presence of dysphagia led to a 1.944-unit increase in the STRONGkids score, and 1.497-unit for STAMP. Additionally, for every unit increase in the STRONGkids score, the length of hospital stay increased by 2.541 days, and hospitalization costs increased by $612.507. Similarly, for every unit increase in the STAMP score, the length of hospital stay increased by 1.571 days, and hospitalization costs increased by $425.595.

**Conclusions:**

Based on decision tree, causal analysis and the actual situation of SNI, the internal structural setup of the STAMP tool is more suitable for screening pediatric patients with JE, making it a more reasonable choice for this purpose when compared to STRONGkids.

## Background

Japanese encephalitis (JE) is an acute infectious disease caused by the Japanese encephalitis virus (JEV), which affects the central nervous system. JE is also a zoonotic disease with high mortality and sequelae rates. The mortality rate peaks at 20%–30%, and among survivors, approximately 30%–50% may endure permanent neurological or psychiatric sequelae such as aphasia, disturbance of consciousness, and limb paralysis [1]. Globally, 24 countries, including China, face the risk of JEV transmission [2]. Presently, apart from Xinjiang, Xizang, and Qinghai, other regions in China are susceptible to JEV transmission. Owing to climate, residents’ habits, and geographical conditions, Chongqing has perennially exhibited a high incidence of JE [3], ranking consistently among the top two provinces in China in terms of incidence rates.

The primary clinical manifestations of JE in children include fever, headache, vomiting, convulsions, and disturbances of consciousness. Some cases may result in neurological sequelae. These sequelae can negatively impact the body’s nutritional reserves and intake, leading to disease-related malnutrition and acute sarcopenia, which are significantly correlated with decreased quality of life, muscle dysfunction, increased complication rates, and mortality [4]. Therefore, research on clinical nutrition management for hospitalized children with JE holds crucial practical significance. Assessing nutrition risk is the initial step in nutrition management, and the evaluation is typically rapid and straightforward. Early application helps in identifying children with low nutritional reserves and poor prognoses. The Screening Tool for Risk of Nutrition in Growth Kids (STRONGkids) and Screening Tool for Assessment of Malnutrition in Pediatrics (STAMP) are commonly employed to evaluate the nutritional risk of children upon admission. Integrating the child’s nutritional status and disease diagnosis helps in determining nutritional risks and potential treatment responses [5]. Until now, research on the nutritional risk of children with JE is non-existent, and the correlation between nutritional risk and clinical outcomes also remains in a blank state.

Although studies have indicated a significant correlation between STRONGkids/STAMP and factors such as hospital stay and hospitalization costs, the precise causal relationship between them remains unclear. Other variables may influence this correlation, but correlation does not imply causation. Although correlation can aid in prediction, it should not be the sole basis for decision-making. An article recently published in the JAMA journal pointed out that clinical research should pay more attention to causality rather than just correlation [6]. To gain a deeper understanding of the relationship between variables and offer guidance for treatment, this study employs causal reasoning to investigate the genuine causal relationship between variables, according to data from nearly 10 years of pediatric JE cases in a tertiary children’s hospital. The objective is to explore the connection between nutritional risk and clinical outcomes, thereby providing a theoretical foundation for scientific nutritional management.

## Methods

### Subjects

We collected data from 198 children diagnosed with JE who were admitted to our research center, a tertiary pediatric hospital and regional pediatric medical center in China, between 2013 and 2023. Diagnosis was established based on the “Diagnostic Criteria for Japanese Encephalitis” (WS214-2008) issued by the Ministry of Health of the People’s Republic of China. Cases that met the epidemiological history, clinical symptoms, signs, and laboratory tests indicative of JE were clinically diagnosed. Cerebrospinal fluid and/or serum samples from clinically diagnosed patients were sent to our hospital’s laboratory, where reported results confirmed the presence of positive immunoglobulin M (IgM) antibodies for JEV.

The exclusion criteria were as follows: 1) discharged against medical advice; 2) corrected gestational age of less than 1 month; 3) presence of other nutrition-related diseases, such as hematologic neoplastic diseases, cerebral palsy, inflammatory bowel disease, short bowel syndrome, endocrine genetic metabolic disease and chronic diarrhea (other disease severity factors that influence the scores of the two NRS tools); 4) hospitalization duration of less than 48 h; 5) guardians unaware of the child’s recent diet and weight changes.

### Nutrition risk screening

The initial step in nutritional management involves screening for nutritional risk, our research adopted two scoring standards: STRONGkids and STAMP. The specific entries and scoring criteria for the two NRS tools are presented in Tables 8 and 9 in Appendix.

STRONGkids consists of four parts: high-risk diseases, subjective clinical assessment, nutritional intake and loss, and weight loss or inadequate gain. A total score of 0 indicates low nutritional risk, while scores of 1 to 3 indicate moderate nutritional risk, and scores of 4 to 5 indicate high nutritional risk (HNR).

STAMP evaluated underlying disease, nutritional intake and differences in weight and height percentiles, and the total score varies from 0 to 9. The score 4 and higher means a HNR, while scores of 0 to 1 indicate low nutritional risk, and scores of 2 to 3 indicate moderate nutritional risk.

### Clinical swallowing function assessment

Clinical swallowing function assessment: In this study, certain children exhibited symptoms of swallowing dysfunction such as disturbance of consciousness, drooling, and chewing difficulties, and these children were categorized as having dysphagia.

### Definition of malnutrition

According to the growth standards set by the WHO, the Anthro and AnthroPlus software are indeed used to calculate Z-scores, as detailed below:

1. For children aged 2 and above: Children meeting any one of the following four criteria were considered to have malnutrition:①BMI-for-Age Z-score < −2;②Underweight: Weight-for-age Z-score < −2;③Growth retardation: Height-for-age Z-score < −2;④Obesity: BMI-for-Age Z-score > 2.
2. For children under 2 years old: Children meeting any one of the following four criteria were considered to have malnutrition:①Underweight: Weight-for-age Z-score < −2;②Growth retardation: Height-for-age Z-score < −2;③Wasting: Weight-for-height Z-score < −2;④Overweight/obesity: Weight-for-height Z-score > 2.

### Data collection

The nutritional risk screening tool STRONGkids and STAMP were administered within 24 h of patient admission, and general information such as the child’s name, gender, age, clinical manifestations, and assessment date were recorded. Following the child’s discharge, their hospital stay and hospitalization costs were documented.

### Statistical analysis

Descriptive statistics were computed using SPSS (Version 27.0) to summarize the general characteristics of the data analyzed in this study. Patient age was reported as the median with interquartile range [Q1, Q3]. Categorical variables were presented as counts and percentages, including sex (female and male, %), disease severity categories (mild, severe, and extremely severe, %), and nutrition risk (HNR and low to moderate nutritional risk [LMNR], %). The association between variables was evaluated using the Spearman correlation coefficient, with a *p*-value below 0.05 considered statistically significant. The Kappa coefficient is used for consistency analysis, and the levels of consistency represented by the Kappa coefficient are: Kappa > 0.8 is considered very good, 0.6 < Kappa ≤ 0.8 is good, 0.4 < Kappa ≤ 0.6 is moderate, 0.2 < Kappa ≤ 0.4 is fair, and Kappa ≤ 0.2 is poor. A *p*-value of < 0.05 indicates statistical significance.

### Decision tree (Machine learning)

The decision tree classification and regression tree (CART) is a commonly used machine learning method widely applied in classification and regression problems [7]. The method involves the construction of a tree-like structure to model and predict data. In a decision tree, each internal node represents a feature or attribute, while each leaf node represents a class or predicted value. The objective is to partition the data into subsets of higher purity by selecting the most discriminative features.

Decision trees offer valuable insights into feature importance. Feature importance involves the evaluation of the purity gains or other indicators acquired when a feature is employed to split data within the decision tree. In this study, we evaluated the significance of features based on their placement in the tree and the Gini coefficient of the splitting point. This evaluation allows for quantifying the contribution of features to model performance, thereby facilitating the comprehension of key features within the data.

### Causal inference using DoWhy

**Fig. 1:**
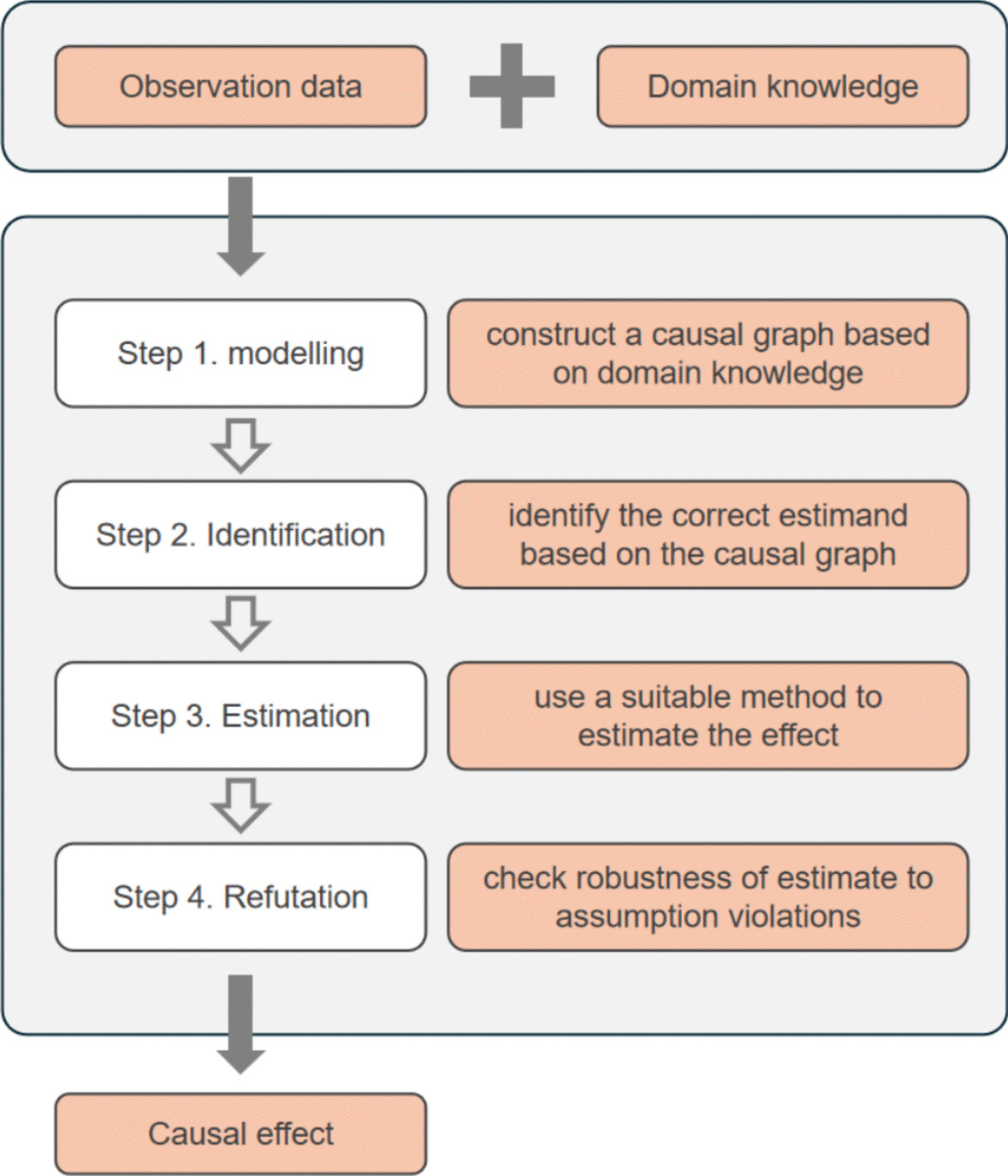
Four-step analysis pipeline in DoWhy

The DoWhy library, a Python tool for causal inference, integrates causal graph models and potential outcome models. In this study, we employed the construction of causal model graphs to quantitatively estimate causal effects and validate causal hypotheses [8]. As illustrated in Fig. 1, this study comprised four steps.

1. Modeling: Constructing a plausible causal graph based on expert prior knowledge regarding the treatment process of JE is a crucial step in causal inference. This entails modeling the relationships between variables of interest and their mutual influences. Such an approach facilitates the visualization of causal relationships between variables, which is essential for analyzing and understanding potential causal pathways. In DoWhy, a causal graph is represented as a directed acyclic graph, where a directed edge from X to Y, denoted as X → Y, indicates that X causes Y, signifying that X is the cause of Y.
2. Identification: The effect to be estimated is identified using criteria such as the backdoor criterion or the frontdoor criterion. The purpose of these criteria is to block non-causal paths in the causal graph.
3. Estimation: During the estimation phase, the model calculates the treatment effect after adjusting for confounding factors. Linear regression is employed for all estimations except during the estimation of clinical outcomes, for which logistic regression is used.
4. Refutation: To validate the results obtained in the third step, three methods are employed: adding a random common cause, utilizing a placebo treatment, and data subset validation. The “random common cause” method involves introducing a common unobserved random variable to the original data to test the model’s sensitivity to unobserved confounding variables. The “placebo treatment” method is similar to cross-validation in predictive modeling. In this method, the actual treatment is replaced with random variables to determine if the entire model contains errors, considering the known causal effects of variables. In the “data subset” method, randomly selected subsets replace the original data to assess the variance of effects generated in the estimation step. If the results of the “random common cause” method do not significantly differ from the estimated effect, the same is expected for the “data subset” method. Conversely, the result of the “placebo treatment” method tends toward zero, indicating that the estimator is robust and that the estimated effect passes all three validation methods.

## Results

### Characteristics of study cohort

**Fig. 2:**
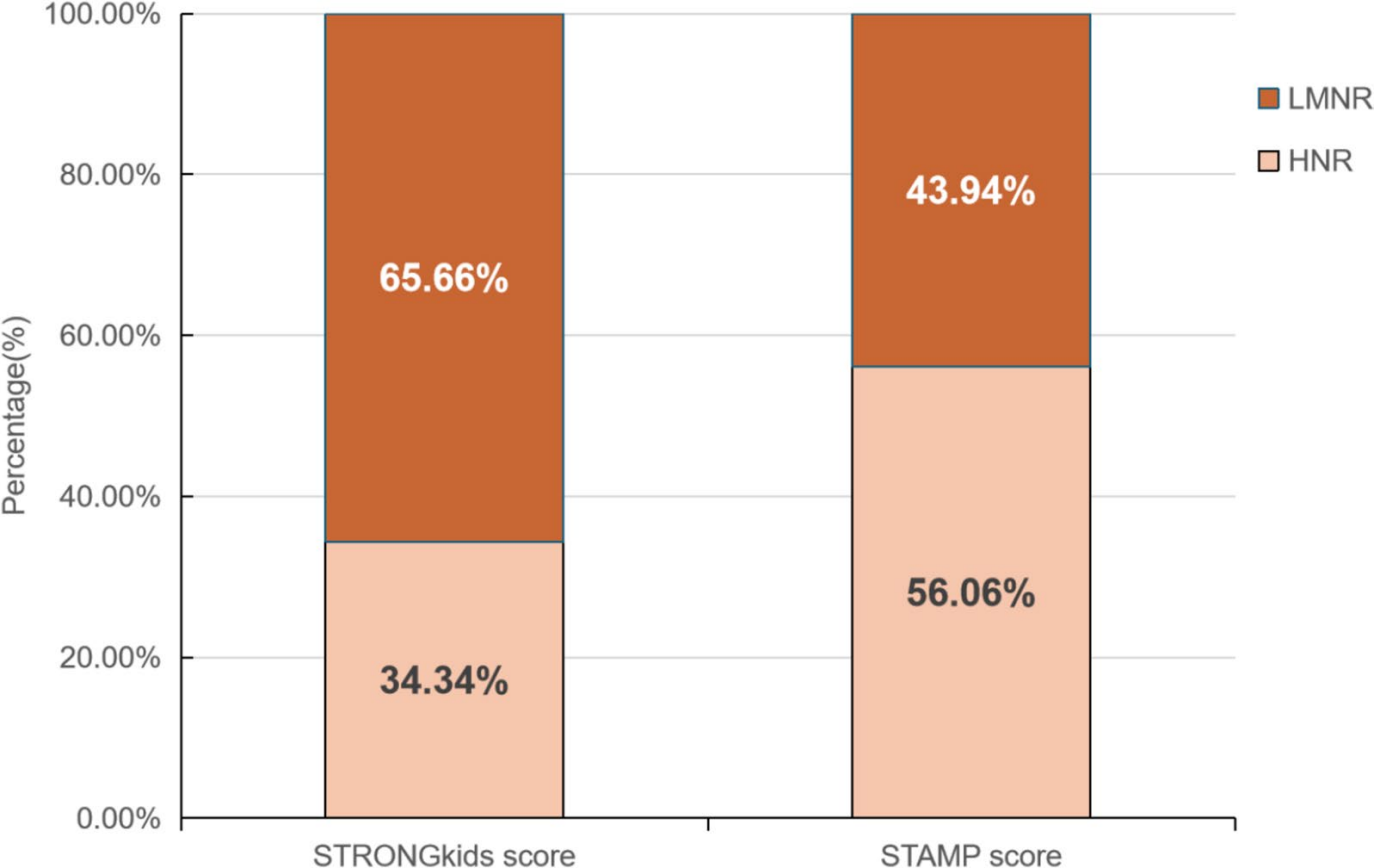
The distribution of HNR and LMNR in STRONGKids scores and STAMP scores

A total of 198 cases, comprising 111 males (56.06%) and 87 females (43.94%), were collected. The median age of the children was 72 months, with an interquartile range of [36, 108]. Owing to our research unit’s specialization in treating severe cases of JE, no mild cases were included in our study. Thus, there were 98 cases of moderate severity (49.50%), 71 cases of severe severity (35.86%), and 29 cases of extremely severe severity (14.64%). The composition ratio of the LMNR group and the HNR group in the two nutritional risk screening (NRS) tools are as follows: 65.66% LMNR and 34.34% HNR for STRONGkids, while 43.94% LMNR and 56.06% HNR for STAMP; as indicated in Table 1 and Fig. 2.

**Table 1:** The general information of the patient

Characteristics	Values
**Age median [Q1, Q3] (months)**	72 [36, 108]
**Sex**	
Female	87 (43.94%)
Male	111 (56.06%)
**Types**	
Moderate	98 (49.50%)
Severe	71 (35.86%)
Extremely severe	29 (14.64%)

The distribution of HNR and LMNR in STRONGKids scores and STAMP scores is depicted in Fig. 2, the results show that HNR in STRONGKids score remains as HNR in STAMP score, but 43 cases of LMNR in STRONGKids score would be classified as HNR in STAMP score.

In terms of consistency in assessing nutritional risk between the two NRS tools, STRONGkids and STAMP showed moderate agreement, with a Kappa value of 0.5815 (*p* < 0.01), 95% CI (0.4810, 0.6820).

The factors influencing children’s STRONGkids scores include the presence of dysphagia, malnutrition upon admission, reduced dietary intake, and weight loss (from onset to screening);while the main factors that influence STAMP scores include the presence of nutrition-related disease diagnoses (dysphagia in this study), weight and height, nutritional intake, as indicated in Tables 2 and 3.

**Table 2:** Proportion of components in comprehensive assessment for STRONGkids score

	Dysphagia	Malnutrition	Nutritional intake loss	Weight loss
Exist	148(74.74%)	20(10.10%)	183(92.42%)	91(45.96%)
Absent	50(25.26%)	178(89.90%)	15(7.58%)	107(54.04%)

**Table 3:** Proportion of components in comprehensive assessment for STAMP score

	Dysphagia	Weight and Height	Nutritional intake
0	50(25.26%)	100(50.5%)	104(52.52%)
1	/	67(33.84%)	/
2	0	/	69(34.85%)
3	148(74.74%)	31(15.66%)	25(12.63%)

In the STAMP score, if there is no nutritional intake at all, it’s worth 3 points. If the nutritional intake is between 25 and 50%, it’s worth 2 points. If there is no change in the nutritional intake or the nutritional intake is more than 50%, it’s worth 0 points

### Correlations between the variables of the study

**Fig. 3:**
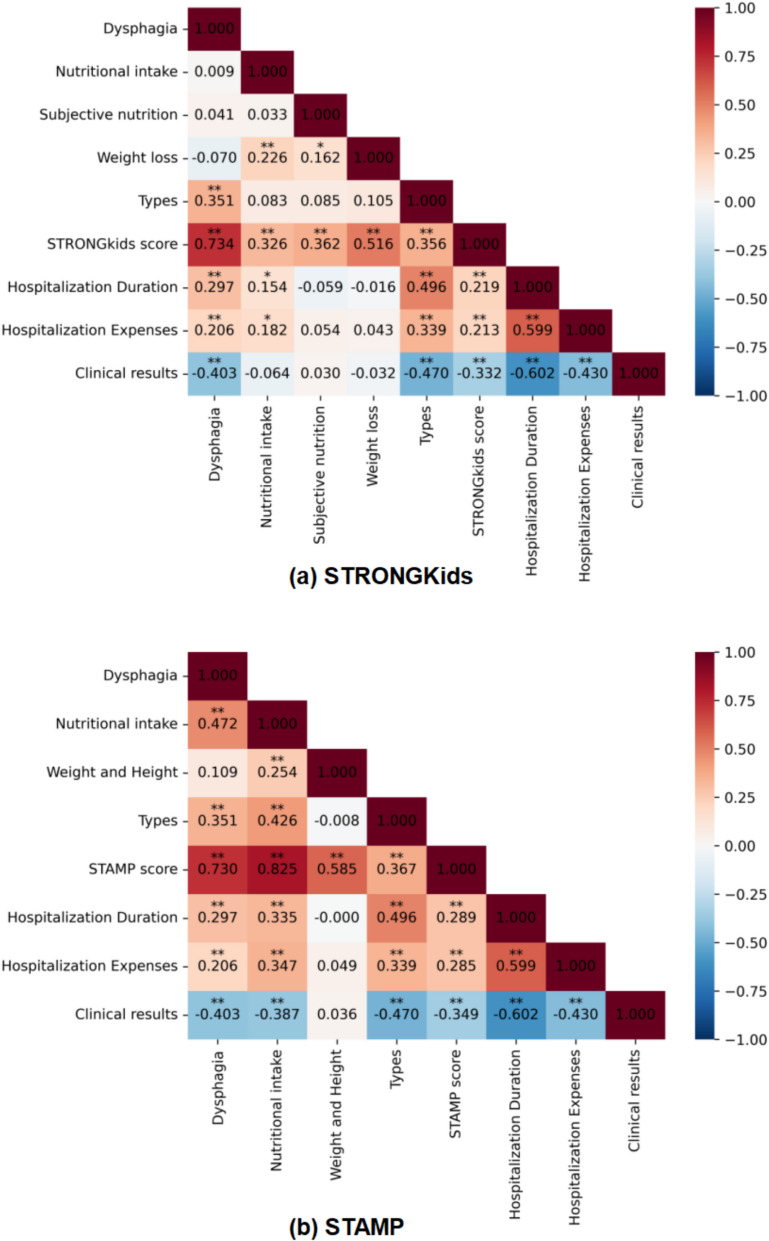
Heatmap of Spearman correlation coefficients among the variables; **a** STRONGkids score; **b** STAMP score (* indicates that the correlation coefficient is statistically significant at *p* < 0.05, ** indicates the correlation coefficient is statistically significant at *p* < 0.01). These coefficients elucidate the relationships among the data for each variable. Notably, factors like dysphagia, subjective nutrition, nutritional intake, and weight loss demonstrate significant correlations with the STRONGkids score, with dysphagia exhibiting the strongest link. Additionally, the STAMP score shows a marked correlation with dysphagia, weight and height, nutritional intake, among which nutritional intake holds the highest correlation

Figure 3 illustrates the heatmap of correlations among variables, with numerical values representing correlation coefficients between two variables. These coefficients describe the relationship within the data of each variable. For example, factors such as dysphagia, subjective nutrition, nutritional intake, and weight loss were all significantly correlated with the STRONGkids score. Among them, dysphagia exhibited the highest correlation coefficient of 0.734 (95% CI [0.660, 0.794], *p* < 0.01).the second most relevant factor is weight loss, with a correlation coefficient of 0.516 (95% CI [0.402, 0.614], *p* < 0.01), and the third is subjective clinical assessment. with a correlation coefficient of 0.362 (95% CI [0.230, 0.481], *p* < 0.01).Meanwhile the STAMP score is significantly correlated with dysphagia,weight and height,nutritional intake, with the highest correlation coefficient being 0.825 (95% CI [0.773, 0.866], *p* < 0.01) for nutritional intake,the second most relevant factor is dysphagia, with a correlation coefficient of 0.730 (95% CI [0.655, 0.791], *p* < 0.01), and the third is weight and height. with a correlation coefficient of 0.585 (95% CI [0.482,0.672], *p* < 0.01). Additionally, clinical results is the only factor that has a negative correlation coefficient with both STRONGkids score and STAMP score, indicating that a higher STRONGkids score or STAMP score correlated with a worse prognosis and a higher likelihood of sequelae.

### Key factors of two NRS tools revealed by decision trees

**Fig. 4:**
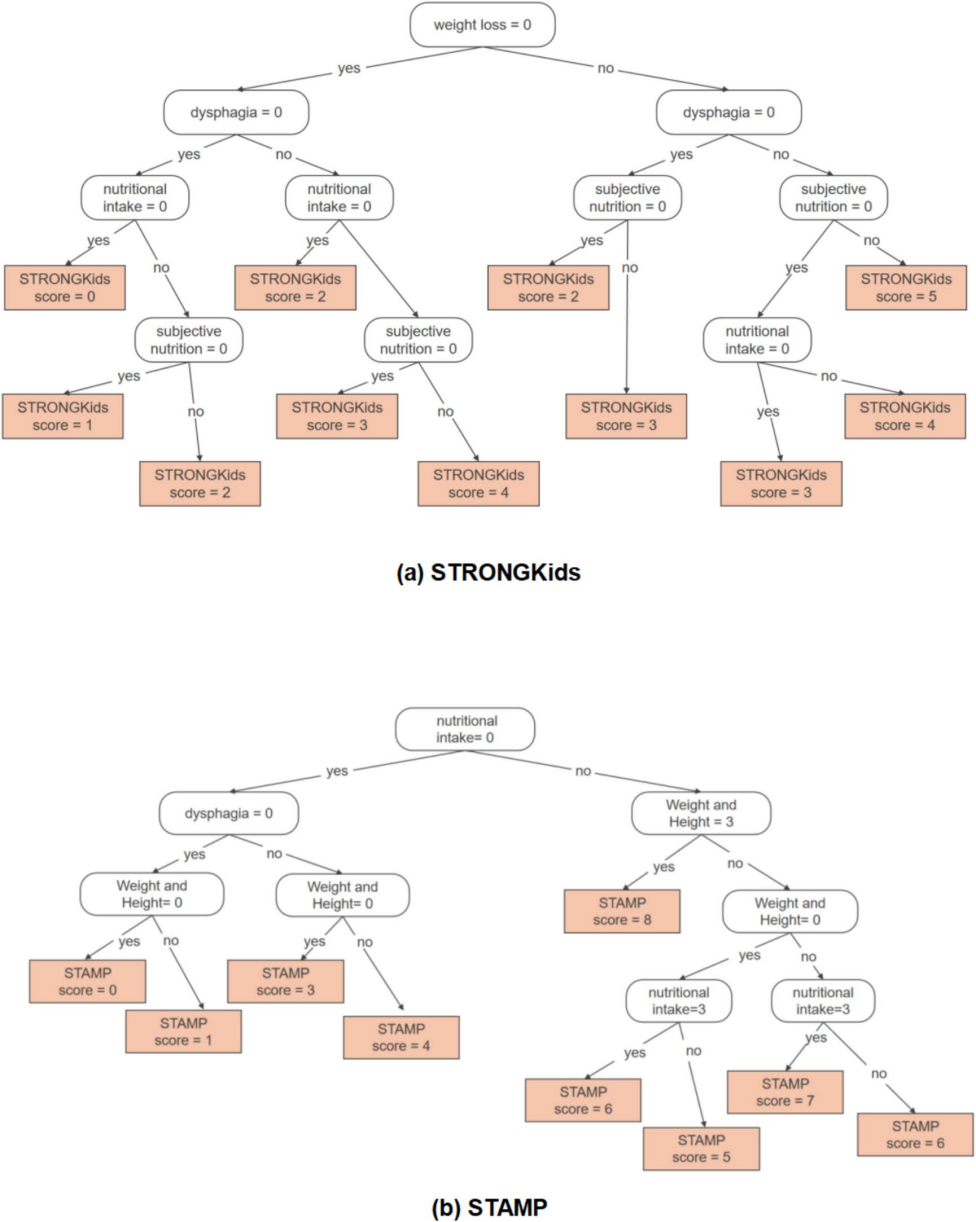
**a** Decision tree of STRONGKids score; **b** Decision tree of STAMP score. Performed an in-depth analysis of two NRS tools utilizing CART to identify the key factors that influence their scores

**Fig. 5:**
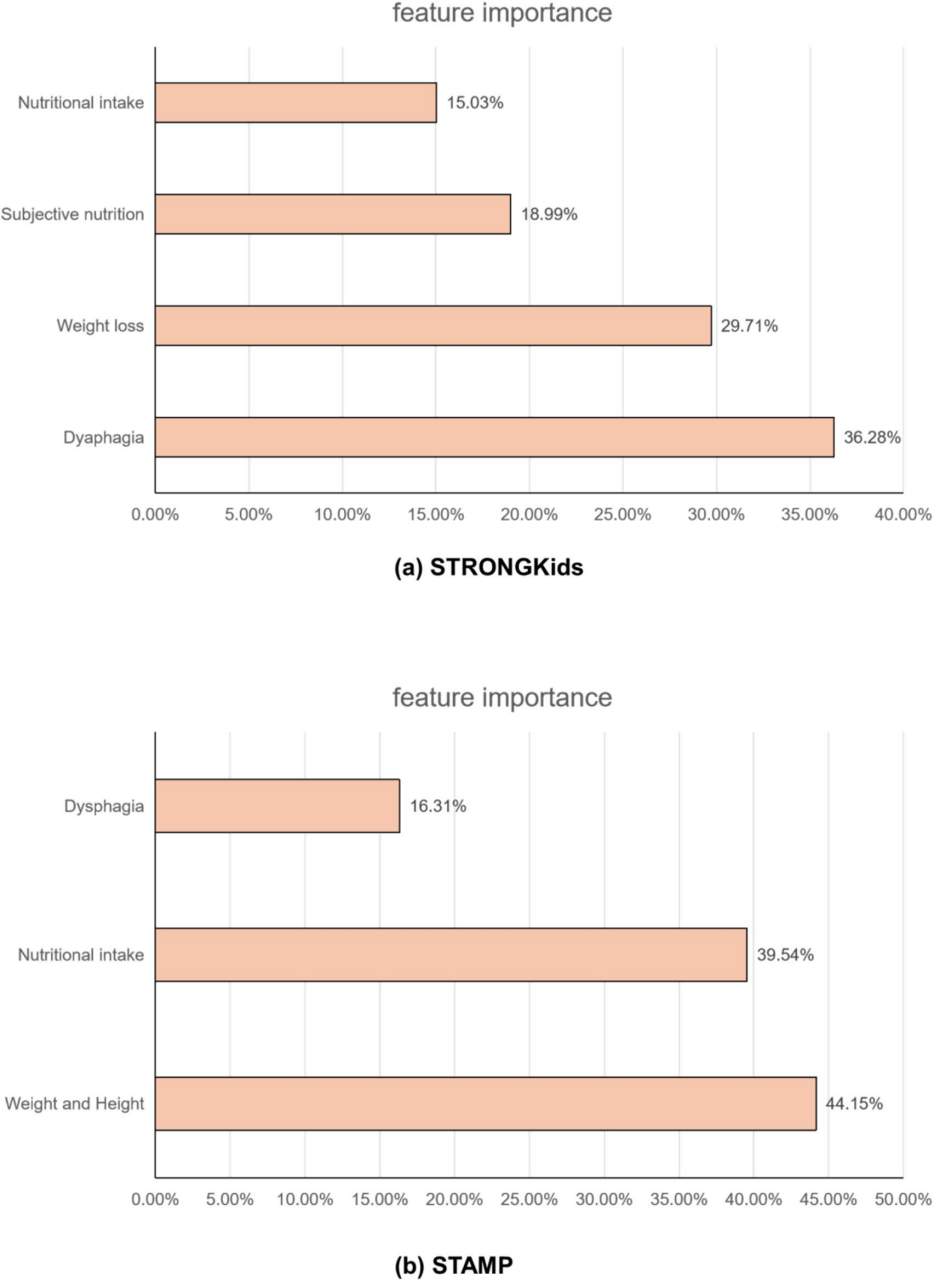
**a** Feature importance of STRONGKids scores, as obtained using a decision tree; **b** Feature importance of STAMP scores, as obtained using a decision tree. This plot demonstrated the significance of each feature in influencing two NRS tools. The analysis revealed that dysphagia was the most significant factors affecting STRONGkids scores, and the Weight and Height was the most significant factors affecting STAMP scores

This study conducted an in-depth analysis of two NRS tools using CART to reveal the primary factors influencing the scores. The decision tree diagram (Fig. 4) illustrates the structure and segmentation rules of the decision tree model, aiding in comprehending the decision-making process at each node. Furthermore, a feature importance analysis was conducted, and a feature importance plot (Fig. 5) was generated. This plot demonstrated the significance of each feature in influencing two NRS tools. The analysis revealed that dysphagia was the most significant factors affecting STRONGkids scores, with a feature importance of 36.28%; and the Weight and Height was the most significant factors affecting STAMP scores, with a feature importance of 44.15%.

### Causality between the variables of the study

**Fig. 6:**
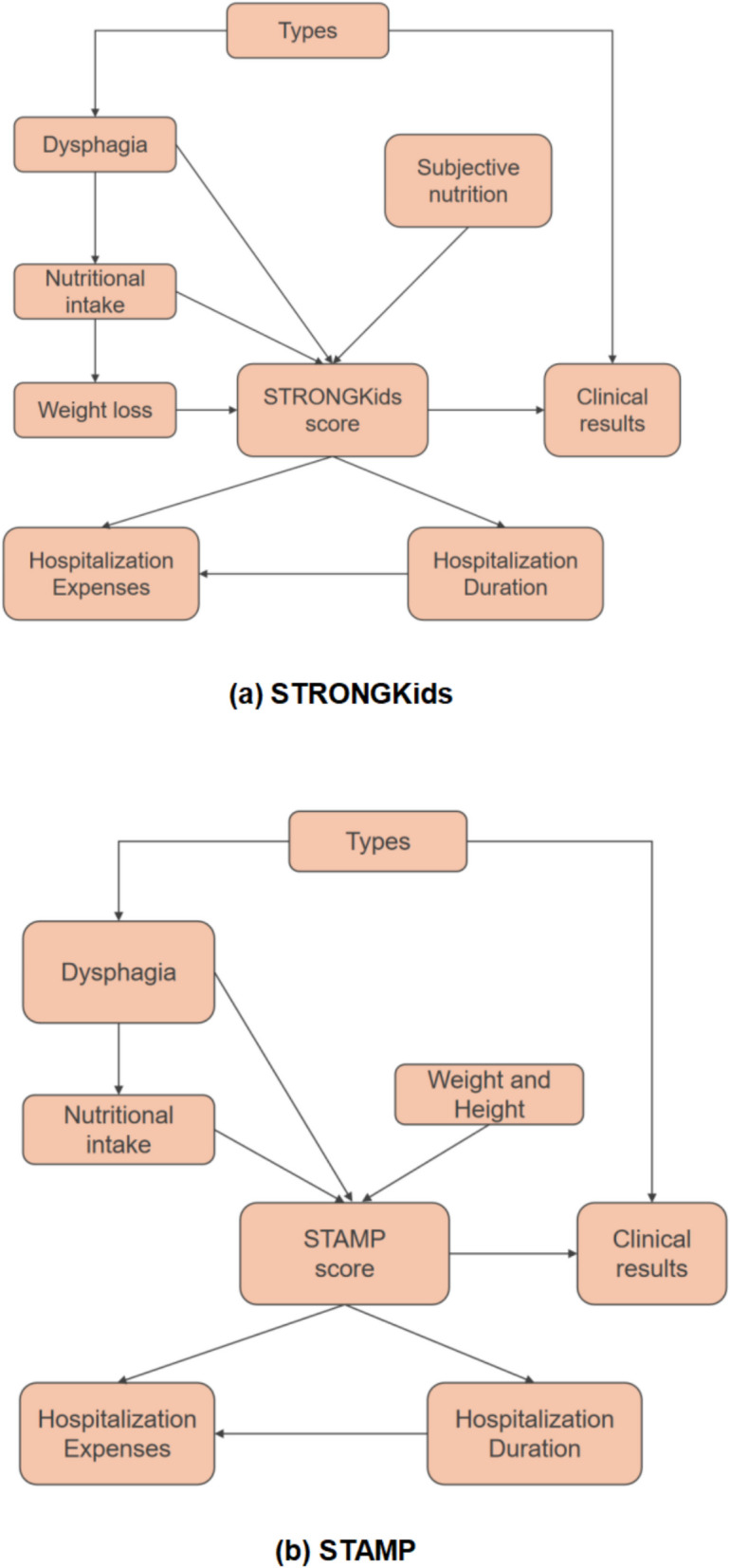
**a** Causal graph of STRONGkids score; **b** Causal graph of STAMP score. In these diagrams, an arrow pointing from X to Y (X — > Y) indicates that X is a causal factor of Y

According to the previous study on correlation, this study explored the causal relationships among highly correlated variables. Figure 6 illustrates the causal inference model employed in this study. Treatment and outcome variables were chosen, and the average treatment effect of the treatment variable on the outcome variable was estimated.

- “Types” indicates the severity of encephalitis, where 0 represents moderate, 1 represents severe, and 2 represents extremely severe.
- “Dysphagia” indicates the severity of the disease, where 0 and 2 respectively represent absence and high-risk disease.
- “STRONGkids score” represents the patient’s STRONGkids score, with a score of 0 indicating low nutritional risk, 1–3 points indicating medium nutritional risk, and 4–5 points indicating HNR.
- “Hospitalization duration” indicates the length of hospital stay.
- “Hospitalization expenses” indicates the expenses incurred during the patient’s hospitalization.
- “Clinical results” represents clinical prognosis, that is, whether the patient has sequelae, where 0 indicates the presence of sequelae, and 1 indicates a good prognosis.

The summarized causal effects (Table 4) indicate changes in outcomes when the treatment was increased by a unit. The estimated causal relationships in this study encompassed types, dysphagia, clinical outcomes, and clinical results in relation to the STRONGkids score. Regarding the causal effects of types and high-risk disease on the STRONGkids score, an increase of one unit in patient type was associated with a 0.515-unit rise in the STRONGkids score, while transitioning from no dysphagia to high risk resulted in a 1.944 unit increase in the STRONGkids score. This suggests that greater severity in types and disease severity (dysphagia) corresponded to higher STRONGkids scores. Furthermore, regarding the impact of the STRONGkids score on clinical outcomes and clinical results, for every unit increase in the STRONGkids score, hospitalization duration increased by 2.541 days, hospitalization expenses increased by $612.507, and clinical results decreased by 0.068 units. This indicates that higher STRONGkids scores correlated with longer hospital stays, higher hospitalization expenses, and poorer prognosis, suggesting a greater likelihood of patients developing sequelae. Similar trends were observed for STAMP score, as shown in Table 5. (An increase of one unit in patient type was associated with a 1.339-unit rise in the STAMP score, while transitioning from no dysphagia to high risk resulted in a 1.497 unit increase in the STAMP score. regarding the impact of the STAMP score on clinical outcomes and clinical results, for every unit increase in the STAMP score, hospitalization duration increased by 1.571 days, hospitalization expenses increased by $425.595, and clinical results decreased by 0.036 units. The results of the three refutation tests are presented in Tables 10 and 11 in Appendix.

**Table 4:** Estimated effect of treatment on outcome of STRONGKids score

Treatment	Outcome	Estimated effect (95%CI)	*p* value
Types Dysphagia	STRONGKids score	0.515 (0.379, 0.670)	8.29e − 6
	STRONGKids score	1.944 (1.759, 2.142)	1.23e − 40
STRONGKids score	Hospitalization duration	2.541 [−0.483, 5.566]	0.101
STRONGKids score	Hospitalization expenses	612.507[89.259,1135.754]	0.022
STRONGKids score	Clinical results	−0.068 (−0.102, −0.048)	0.006

**Table 5:** Estimated effect of treatment on outcome of STAMP score

Treatment	Outcome	Estimated effect (95%CI)	*p* value
Types	STAMP scores	1.339(0.982, 1.694)	1.21450478e-05
Dysphagia	STAMP scores	1.497(1.382, 1.627)	8.28124887e-22
STAMP scores	Hospitalization duration	1.571[0.254, 2.889]	0.01966975
STAMP scores	Hospitalization expenses	425.595[200.810, 650.381]	0.00024703
STAMP scores	Clinical results	−0.036 (−0.054, −0.021)	0.002

### The cost and duration of specialized nutritional intervention (SNI) for different levels of nutritional risk

This study also analyzed the cost and duration of nutritional interventions for children with different nutritional risks. The results showed that children with HNR required higher costs and longer durations for nutritional interventions, while children with LMNR had relatively lower costs and shorter durations for nutritional interventions.

In STAMP, there were significant differences in hospitalization expenses, days of nutritional therapy, and nutritional therapy expenses between HNR and LMNR (*P* < 0.05). However, in STRONGkids, there were no differences in hospitalization days, hospitalization expenses days of nutritional therapy, and nutritional therapy expenses between HNR and LMNR, as indicated in Table 6.

**Table 6:** The cost and duration of SNI for different levels of nutritional risk

NRS Tool	Risk Group	Average Hospitalization Expenses($)	*p*-value	Average Hospitalization Duration(days)	*p*-value	Average SNI Expense($)	*p*-value	Average SNI Days(days)	*p*-value
STAMP	HNR	5208.043063	*p* < 0.05	26.414414	*p* < 0.05	473.454955	*p* < 0.05	1.711712	*p* < 0.05
	LMNR	3114.684023		17.482759		18.218391		0.126437	
STRONGKids	HNR	4770.304118	*p* > 0.05	24.294118	*p* > 0.05	508.522500	*p* > 0.05	1.897059	*p* > 0.05
	LMNR	4036.073923		21.546154		150.453615		0.553846	

### The composition ratio of SNI of the LMNR group and the HNR group

Table 7 shows that there were 111 cases of HNR in STAMP, and 68 in STRONGkids, but only 25 cases received SNI without distinguishing nutritional risks. The composition ratio of SNI in the 25 cases in LMNR group and the HNR group of the two NRS tools are as follows: 56% (14/25) LMNR and 44% (11/25) HNR for STRONGkids, while 16% (4/25) LMNR and 84% (21/25) HNR for STAMP.In STAMP, 18.91% (21/111) of children with HNR received SNI.

**Table 7:** The composition ratio of SNI of the LMNR and HNR

NRS Tool	Nutrition Risk Group	SNI *n* = 25	No SNI *n* = 173	*P*-value
STAMP score	HNR *n* = 111	*n* = 21	*n* = 90	*p* < 0.05
	LMNR *n* = 87	*n* = 4	*n* = 83	
STRONGKids score	HNR *n* = 68	*n* = 11	*n* = 57	*p* > 0.05
	LMNR *n* = 130	*n* = 14	*n* = 116	

For each NRS tool, the Chi-square test was performed to determine the association between the acceptance of SNI and the nutrition risk groups (HNR and LMNR). For the STAMP tool, the results showed a significant association (*p* < 0.05), indicating that the likelihood of receiving SNI is significantly different between the HNR and LMNR groups. In contrast, for the STRONGkids tool, the association was not significant (p > 0.05), suggesting no significant difference in the acceptance of SNI between the HNR and LMNR groups.

## Discussion

JE is predominantly distributed in Southeast Asia and the Western Pacific region. In China, the disease is mainly concentrated in Chongqing, Sichuan, and Guizhou. Since 1968, the implementation of inactivated Vero cell-derived vaccines, attenuated live vaccines, and live chimeric vaccines has resulted in a reduction in JE outbreaks and a significant decrease in incidence compared with the case in previous years. However, owing to the high disability rate, the burden of JE remains substantial [9]. The majority of JE cases occur in children [10], and the mortality and disability rates are notably high for severe and extremely severe cases, which significantly impacts the quality of life of affected children. As the length of hospital stay increases, the incidence of malnutrition in children with JE gradually rises. A previous study [11] by our team demonstrated that the incidence of malnutrition in children with severe JE increased by 21.1% after one month of hospitalization compared with the initial stage. The incidence of inadequate feeding was notably high, with more than two-thirds of children experiencing a weight loss of over 5% within one month, indicating significant weight loss and a heightened risk of protein-energy malnutrition. This trend is attributable to JEV-induced damage to the nervous system, resulting in swallowing difficulties and inadequate intake, consequently leading to insufficient nutritional support and subsequent weight loss. Malnutrition may trigger the gradual depletion of body organs, including a reduction in myocardial and diaphragmatic muscles, resulting in cardiopulmonary insufficiency, disease exacerbation, and an increase in complications and mortality. Therefore, addressing the nutritional concerns of children with JE is crucial.

In this study, we found that the incidence of malnutrition upon admission was 10.1%, which is similar to the malnutrition rate among Chinese children in 2019 [12]. This similarity suggests that the incidence of malnutrition upon admission in children with JE is similar to that in the healthy population, indirectly indicating that malnutrition alone may not render children more susceptible to JEV.

Several studies have highlighted a correlation between nutritional risk and clinical outcomes. However, there is currently no globally standardized pediatric nutritional risk assessment tool. Most pediatric nutrition tools developed internationally, such as STRONGkids, Pediatric Yorkhill Malnutrition Score, and Pediatric Malnutrition Assessment Screening Tools, primarily serve as malnutrition risk screening tools [13, 14]. They are based on the principles of nutritional screening tools outlined by the European Society for Parenteral and Enteral Nutrition (ESPEN) and can be temporarily employed as proxies for “nutritional risk” tools in clinical settings. Clinical experience has shown that the specificity and sensitivity of STRONGkids and STAMP are high, and the two NRS tools can ensure the early identification of children with nutritional risks. Nutritional risk upon admission has been linked to clinical outcomes [15–17]. To gain a deeper understanding of the causal links between variables and offer insights into nutritional therapy, we conducted this study, building upon prior research. Moreover, nutritional risks and the factors influencing them, along with clinical outcomes, differ across various diseases. Presently, research on the nutritional risks of children with JE is limited, and the correlation and causal relationship between nutritional risks and clinical outcomes remain largely unexplored.

In our study, through the statistical method of heatmap, it is known that dysphagia demonstrated the highest correlation with the STRONGkids score, followed by weight loss, meanwhile. nutritional intake demonstrated the highest correlation with the STAMP score, followed by dysphagia. However, correlation does not imply causation. Furthermore, we employed decision tree analysis for an in-depth exploration of the two NRS tools to reveal its key influencing factors. Additionally, a feature importance plot was generated to illustrate the significance of each feature to the two NRS score. Our results indicated that “dysphagia” was the most critical factor affecting the STRONGkids score; while “weight and height” was the most critical factor affecting the STAMP score. Subsequent causal inference further confirms the overall impact of dysphagia on the scores of the two tools (Table 6). The difference in the results of the two NRS tools is related to the different scoring of their respective parameters during the evaluation different tools have different focuses. We have found that the STAMP tool gives greater emphasis to the actual nutritional status of pediatric patients upon admission(weight, height and nutritional intake); whereas the STRONGkids tool focuses more on the factors influencing nutritional status(weight loss history and whether dysphagia exists), but these factors do not fully capture the current extent to which actual nutritional intake is affected (even in the absence of dysphagia, illnesses can still lead to inadequate food intake;even in the absence of weight loss, the patient could also be in a condition of nutritional deficiency prior to the onset of the illness). Hence, STRONGkids seems inadvertently overlook this part of JE pediatric patients, inaccurately assigning them to the LMNR. Conversely, the STAMP tool demonstrates greater proficiency in pinpointing JE pediatric patients who genuinely face high nutritional risk. In addition, the consistency between STRONGkids and STAMP also showed inadequate agreement.

For the purpose of further substantiating this hypothesis, we regard the 25 cases who had undergone SNI after consultation with the clinical nutrition department as the “gold standard for HNR”; the results indicated that STAMP exhibited higher sensitivity in identifying authentic JE pediatric patients with HNR (Table 7), which matched our predictions. From another perspective, the data from STRONGkids indicates that there is no difference in the proportion of pediatric patients with JE receiving SNI between the LMNR group and the HNR group (*P* > 0.05), which renders the NRS clinically insignificant;however,in STAMP, the proportion of SNI in the HNR group is higher than that in the LMNR group (*P* < 0.05). Therefore, the STAMP tool is a better choice for NRS of pediatric patients with JE,so that they can receive SNI in a higher percentage.

What we are more concerned about is the impact of different NRS groups and different NRS tools on clinical outcomes. A closer inspection of the heatmap revealed that clinical outcomes were the only indicator with a negative correlation coefficient. This implies that a higher NRS score was associated with poorer clinical outcomes and a greater likelihood of developing sequelae. This finding aligns with clinical theories suggesting that more severe cases of JE in children often involve a higher probability of dysphagia, the less nutrition intake, the lower weight score, and the higher NRS score, resulting in a worse prognosis for the children. To further substantiate the causal relationship, we developed a causal derivation diagram to estimate the causal effects between variables. We utilized the NRS scores as the causal variable to evaluate its impact on clinical outcomes and results. The analysis revealed that the trends of the two NRS scores related to clinical outcomes are consistent, i.e., for every one-unit increase in the NRS scores, the length of hospital stay is longer, the hospitalization cost is higher, the prognosis is worse, and the patients are more likely to have sequelae. Furthermore, the HNR group in STAMP demonstrated elevated hospitalization expenses, prolonged hospitalization duration, and increased days and expenses related to SNI compared to the LMNR group. Notably, such disparities were completely absent in STRONGkids, reinforcing the notion that STAMP is superior in accurately identifying HNR patients and anticipating clinical outcomes (Table 6).

The primary objective of this study is to demonstrate the impact of nutritional risk on the clinical outcomes and results of children with JE, thereby enhancing clinicians’ awareness of the nutritional status of JE-affected children who are at nutritional risk, particularly those with reduced dietary intake, which are major factors contributing to HNR in STAMP. Adequate nutritional support has the potential to mitigate nutritional risks, promote liver protein synthesis, facilitate the synthesis of neurotransmitters, and aid in the restoration of nerve function. Furthermore, it can enhance the body’s immunity, effectively prevent secondary infections, and ameliorate symptoms of refractory cerebral edema without exacerbating cerebral edema or interfering with blood glucose, plasma osmolarity, or intracranial pressure homeostasis [18–20]. However, implementing nutritional intervention measures in China is challenging owing to the occurrence of regional disparities and varying levels of awareness among medical professionals and families. In our study, both of the two NRS indicated a low proportion of specialized nutritional intervention.Also,recent multicenter studies in China have revealed that clinicians often lack sufficient awareness of nutritional needs in children with neurological impairments [21], leading to inadequate attention and non-standardized intervention measures. Therefore, gradually establishing a standardized multidisciplinary nutritional management process (involving doctors, nurses, dieticians, pharmacists, and rehabilitation therapists) in the routine clinical diagnosis and treatment of children with neurological impairments, such as JE, is crucial. This holistic approach can enhance the nutritional status, clinical outcomes, and overall results of these children.

## Conclusions

The incidence of HNR was relatively high among children with JE,and HNR screening scores were associated with prolonged hospitalization durations, increased hospitalization expenses, poorer clinical outcomes, and a greater likelihood of sequelae among children affected by JE. STAMP is a suitable tool for JE. In the future, clinical efforts, enhancing communication and training among clinicians, nutritionists, and other relevant disciplines, while continually monitoring nutritional risks and devising personalized nutritional plans, is crucial. This initiative will enable the gradual establishment of a standardized nutritional management protocol for children with JE.

## Data Availability

The data that support the findings of this study can be obtained from the corresponding author upon reasonable request.

## Appendix

**Table 8:** STRONGkids nutritional risk screening score form

Assessment items	Content of nutritional risk assessment	Score
Subjective clinical assessment	Is the patient in a poor nutritional status judged by subjective clinical assessment (diminished subcutaneous fat and/or muscle mass and/or hollow face)?	Good(0) Poor(1)
High risk disease	Anorexia nervosa, Burns, Bronchopulmonary dysplasia (maximum age 2 years), Celiac disease, Cystic fibrosis, Dysmaturity/prematurity (corrected age 6 months),Cardiac disease, chronic Infectious disease (AIDS), Inflammatory bowel disease, Cancer, Liver disease, chronic, Kidney disease, chronic, Pancreatitis, Short bowel syndrome, Muscle disease, Metabolic disease, Trauma, Mental handicap/retardation, Expected major surgery, Not specified (classified by doctor)	Yes(2) No(0)
Nutritional intake and losses	Are one of the following items present? Excessive diarrhoea (≥ 5 per day) and/or vomiting (> 3 times/day) the last few days? Reduced food intake during the last few days before admission (not including fasting for an elective procedure or surgery)? Pre-existing dietetically advised nutritional intervention? Inability to consume adequate intake because of pain?	Yes(1) No(0)
Weight loss or poor weight gain	Is there weight loss or no weight gain (infants < 1 year) during the last few weeks/months?	Yes(1) No(0)

The total score of the STRONGkids assessment is 5 points, with 0 points indicating low nutritional risk, 1–3 points indicating medium nutritional risk, and 4–5 points indicating high nutritional risk

**Table 9:** STAMP nutritional risk screening score form

Assessment items	Score
Diagnosis (Does the child have a diagnosis that has any nutritional implications?)	
Definitely	3
Possibly	2
No	0
Nutritional intake (What is the child’s nutritional intake?)	
None	3
Recently decreased/poor	2
No change/good	0
Weight and height (Use a growth chart or the centile quick reference tables to determine the child’s measurements)	
> 3 centile spaces/ ≥ 3 columns apart (or weight < 2nd centile)	3
> 2 centile spaces/ = 2 columns apart	1
0 to 1 centile spaces/columns apart	0

A total score of 4 or above indicates high nutritional risk, 2–3 points indicate medium nutritional risk, and 0–1 points indicate low nutritional risk

**Table 10:** Refutation results of STRONGkids score

Treatment	Outcome	Estimated	Refutation results		
			Random Common cause	Placebo treatment	Data subsets validation
Types	STRONGkids score	0.515	0.514	-0.001	0.516
Dysphagia	STRONGkids score	1.943	1.943	-0.011	1.945
STRONGKids score	Hospitalization Duration	2.541	2.543	-0.045	2.541
STRONGKids score	Hospitalization Expenses	612.507	612.279	9.972	615.910
STRONGKids score	Clinical results	-0.068	-0.068	-0.000	-0.067

**Table 11:** Refutation results of STAMP score

Treatment	Outcome	Estimated	Refutation results		
			random common cause	Placebo Treatment	Data subsets validation
Dysphagia	STAMP scores	1.497	1.498	0.004	1.496
Types	STAMP scores	1.339	1.339	0.015	1.341
STAMP scores	Hospitalization duration	1.571	1.574	0.066	1.528
STAMP scores	Hospitalization expenses	425.595	425.453	4.030	433.399
STAMP scores	Clinical results	-0.036	-0.036	-0.000	-0.036

## Abbreviations

JE: Japanese encephalitis
STRONGkids: Screening Tool for Risk of Nutrition in Growth Kids
STAMP: Screening Tool for Assessment of Malnutrition in Pediatrics
HNR: High nutritional risk
LMNR: Low and moderate nutritional risk
JEV: Japanese encephalitis virus
ESPEN: European Society for Parenteral and Enteral Nutrition
CART: Classification and regression Tree
ATE: Average treatment effect
NRS: Nutritional risk screening
